# *Leishmania infantum* Seroprevalence in Cats From Touristic Areas of Italy and Greece

**DOI:** 10.3389/fvets.2020.616566

**Published:** 2020-12-11

**Authors:** Simone Morelli, Mariasole Colombo, Dimitris Dimzas, Alessandra Barlaam, Donato Traversa, Angela Di Cesare, Ilaria Russi, Roberta Spoletini, Barbara Paoletti, Anastasia Diakou

**Affiliations:** ^1^Faculty of Veterinary Medicine, University of Teramo, Teramo, Italy; ^2^School of Veterinary Medicine, Aristotle University of Thessaloniki, Thessaloniki, Greece; ^3^Department of Sciences of Agriculture, Food and Environment, University of Foggia, Foggia, Italy

**Keywords:** *Leishmania infantum*, cats, seroprevalence, Italy, Greece, zoonosis

## Abstract

Leishmaniosis by *Leishmania infantum* is a major zoonotic Vector-Borne Disease (VBD) in terms of geographic distribution, pathogenicity and zoonotic potential. While dogs are the main reservoir of *L. infantum*, the infection in cats is poorly understood although increasingly reported from enzootic and non-enzootic areas. The Mediterranean basin is a key area for leishmaniosis and includes touristic spots that require continuous surveillance for VBDs in consideration of the growing tendency of tourists to travel with their pets. This study evaluated *L. infantum* seroprevalence in cats living in selected touristic localities of Italy and Greece. A total of 269 cat serum samples from three Sites i.e., 76, 40, and 153 from Adriatic Coast of Abruzzo, Italy (Site A), Giglio Island, Tuscany, Italy (Site B), and Mykonos Island, Greece (Site C), respectively, were included in the survey. Sera samples were subjected to an indirect immunofluorescence antibody assay for the detection of anti-*L. infantum* specific IgG. Associations between possible risk factors and seropositivity to *L. infantum* were statistically evaluated. Antibodies against *L. infantum* were detected in eight out of 269 (3.0%) cats tested i.e., 4/76 (5.3%), 1/40 (2.5%), and 3/153 (2.0%), from sites A, B, and C, respectively. A statistical association between anti-*L. infantum* antibodies and cohabitation with dogs was shown. This study indicates that feline populations living in the examined Italian and Greek touristic areas are exposed to *L. infantum* and that they may contribute to the circulation of *L. infantum*, enhancing the risk of infection for dogs and humans.

## Introduction

Leishmaniosis caused by the protozoan parasite *Leishmania infantum* is a Vector-Borne Disease (VBD) of major veterinary and public health concern in the Mediterranean Basin (1, 2), where phlebotomine sandflies of the genus Phlebotomus transmit *L. infantum* to a vertebrate host during blood meal (3). Accordingly, geographic spread, epizootiology, and epidemiology of *L. infantum* are strictly associated with the distribution of these vectors (4, 5).

Current climate changes enhance the reproduction and spread of sandflies and, consequently, promote the rate of *L. infantum* transmission to a wide range of vertebrates, including humans (6, 7). Recent records have shown a rise of infection rates in companion animals (8–10) and wildlife (11–13). Also, autochthonous cases of infection by *L. infantum* in people are reported throughout Europe, including Italy and Greece (14). Southern Europe in particular is regarded as a high-burden area for human visceral leishmaniosis due to *L. infantum* (1).

Dogs are considered the most important domestic reservoir of *L. infantum* (15) while the role of cats as potential reservoir of this parasite requires further corroboration (16). Although cats are in general considered resistant to *L. infantum* (17), they may be infected and potentially act as a source of infection for phlebotomine sandflies (18, 19). In particular, infected cats may successfully pass *L. infantum* to *Phlebotomus perniciosus* in which the parasite further continues its development (20, 21). Additionally, different studies have documented that *L. infantum* circulates among feline populations of Mediterranean basin e.g., Greece, Portugal, Turkey, Cyprus, Spain, and Italy, (22–28). Case reports in cats imported from enzootic areas or traveling with their owners in such localities have shown that feline leishmaniosis may be introduced in non-enzootic countries (29, 30). This latter feature is important in regions with intense movements and travels of animals either with their owners (e.g. tourism), or in the frame of adoption programs of stray animals (animal rights associations activity).

Cats are exposed to *L. infantum* in Italy (28) and Greece (31), which are indeed key epizootiological hubs. Touristic travels are particularly intense in these countries and this is of importance considering the substantial correlation between pet movements and spread of *L. infantum* from enzootic to free areas (10, 31, 32). In such areas a continuous surveillance of the epizootiology and distribution of feline leishmaniosis is pivotal, toward the protection of animal and human health. Thus, the aim of the present study was to evaluate the exposure to *L. infantum* in cats from touristic localities of Italy and Greece, and to investigate possible risk factors associated with the seropositivity in feline populations.

## Materials and Methods

### Animals and Study Areas

Overall, 269 cats (116 in Italy and 153 in Greece) living in three touristic areas were included in the study: 76 cats from Adriatic Coast of Abruzzo, Italy (Site A), 40 cats from Giglio Island, Tuscany, Italy (Site B), and 153 cats from Mykonos Island, Greece (Site C). Detailed information about sex, age, and lifestyle was recorded for each cat. Consent for sample collection and screening for leishmaniosis was obtained from the animal owners or the local municipality authorities and animal rights association authorities, as applicable.

### Sampling and Serology

Blood samples were collected individually in tubes without anticoagulant and centrifuged after clot formation for serum separation and collection. Sera were subjected to an indirect immunofluorescence antibody test (IFAT) for the detection of specific IgG against *L. infantum*. Commercially available slides coated with promastigotes (MegaFLUO Leish—Megacor Diagnostik GmbH) and anti-cat IgG conjugate (FLUO FITC anti-cat IgG conjugate) were used, following the instructions provided by the manufacturer. The cut-off dilution of 1:80 was applied, as indicated in the LeishVet guidelines^1^.

### Statistical Analysis

A statistical analysis was carried out using GraphPad Prism 8 (GraphPad Software, LLC). The Fisher's exact test was used to evaluate the eventual significant associations (*p* ≤ 0.05) between exposure to *L. infantum* and possible risk factors (age, sex, lifestyle, cohabitation with other animals).

Cats were divided in two age classes i.e., aging <36 months old (n. 128), and ≥36 months (n. 141). One hundred and forty cats were male and 129 were female. Overall, 45 cats were housed indoor and were not allowed to go outside, while 224 had constant outdoor access or lived permanently outdoor.

## Results

Overall, antibodies against *L. infantum* were detected in 8/269 cats (3.0%; 95% CI ±2.0) i.e., 4/76 (5.3%; 95% CI ±5.0) from site A, 1/40 (2.5%; 95% CI ±4.5) from site B, and 3/153 (2.0%; 95% CI ±2.2) from site C.

The Fisher's exact test revealed a statistically significant (*p* < 0.05) association between cat seropositivity and cohabitation with dogs (*p* = 0.0360). According to the statistical analysis no other relevant associations were found. The number of seropositive and seronegative cats for each possible risk factor is detailed in Table 1. The median age of both seropositive and seronegative cats was 36 months.

**Table 1:** Number and percentage of cats seropositive and seronegative at IFAT (cut-off dilution 1:80) for *Leishmania infantum* antibodies for each possible risk factor considered in the present study.

	**Seropositive**	**Seronegative**	***P*-value**
	**n/tot (%)**	**n/tot (%)**	
Male	3/140 (2.1)	137/140 (97.9)	0.4862
Female	5/129 (3.9)	124/129 (96.1)	
Outdoor	7/224 (3.1)	217/224 (96.9)	>0.9999
Indoor	1/45 (2.2)	44/45 (97.8)	
<36 m	3/128 (2.3)	125/128 (97.7)	0.7251
≥36 m	5/141 (3.5)	136/141 (96.4)	
Cohabitation with dogs	3/27 (11.1)	24/27 (88.9)	0.0360
No cohabitation with dogs	5/242 (2)	237/242 (97.9)	

*Associations with P-values of < 0.05 were considered statistically significant*.

## Discussion

The data generated in the present study add new information on the exposure to *L. infantum* of cat populations in the examined areas of Italy and Greece.

The overall seroprevalence (3.0%) recorded is in line with values registered in central Spain (1.3–3.2%), Portugal (2.8%) and, very recently, throughout Italy (3.3%) (28, 33, 34). A recent systematic review with meta-analysis of the literature of the last two decades has indicated higher seroprevalence rates in cats from these countries i.e., 11.0% in Greece and 24.0% in Italy (2). Indeed, studies are difficult to compare when different molecular/serological techniques are used, while differences in the prevalence rates could also be attributed to the selection of the sample population (e.g., age, lifestyle, and number of cats), to the selected antibody titer cut-off and to the sampling areas. In fact, IFAT is the most widely used method and, although different values have been applied in different studies (34–39), a cut-off of 1:80 is recommended in the LeishVet group guidelines (see text footnote 1).

The seroprevalence herein detected in Site A (5.6%) is higher than the rates registered recently in the same region (27). This may be attributed to the fact that 3/4 (75.0%) of the positive cats from Site A cohabited with dogs, as this was identified as a statistically significant risk factor by the statistical analysis in the present study. Accordingly, a recent study has demonstrated that a close contact with infected dogs can lead to high seroprevalence rates in cats (40). Nevertheless, this association should be interpreted with caution, because (i) it is generally accepted that the risk of infection does not increase in animals or humans cohabiting with infected dogs, but rather if a high seroprevalence occurs in local dog population and (ii) the parasitological status of dogs cohabiting with positive cats of this study was unknown. Another explanation could be that only cats living in coastal areas of Site A have been examined, while in the previous survey cats were sampled in both mountainous and coastal areas (27). Indeed, altitude and cold temperatures have a negative influence on the biology of *L. infantum* vectors (41, 42). Moreover, some efficient vectors of *L. infantum* e.g., *Phlebotomus neglectus*, find optimal habitat conditions at <1,000 m from the seashore (42, 43). The higher prevalence rates obtained in cats from Abruzzo 15 years ago by IFAT (16.3%) (44), can be explained by the low cut-off value (1:40) which has most likely overestimated the results.

The present data are the first generated on the exposure to *L. infantum* of feline populations living in Site B, a popular touristic island belonging to Tuscany regional territory. The prevalence rate recorded is in line with that detected in a previous study carried out in continental Tuscany in early 2000's (0.9%), (45). Importantly, the single cat that seroreacted was locally born and never moved to other areas. This confirms the presence of the pathogen on the island, which is not surprising, given that Tuscany is a known enzootic area of canine leishmaniosis (46). It should be also considered that Giglio island is a dog-friendly island for tourists^2^ and dogs traveling with their owners from enzootic areas of continental Italy can constantly contribute to the circulation of *L. infantum* among the local fauna. This result thus represents a potential alarm bell ringing for pet populations for the near future.

In a recent study on feline VBDs performed in continental and insular Greece, no *L. infantum* seropositive cats were detected in Mykonos Island (31), a fact that could be attributed to the small size of sampled cats. Therefore, the present results provide new information for this geographic region. In fact, in previous studies in Greece, specific antibodies to *Leishmania* spp. in cats have been detected in Crete Island (14.7%) and in continental Greece i.e., Thessaloniki (3.8%), Athens (8.3%), Thessaly and Macedonia regions (10%) (22, 31, 47). As suggested for Italy, the circulation of *L. infantum* in Site C could have been favored by sociological factors. Indeed, in Greek Islands (including Site C) numerous colonies of stray cats managed by charities and animal welfare organizations arrange adoptions and re-homing of cats^3^ (31). Nonetheless, these commendable initiatives may further concur to the spread of feline leishmaniosis, because cats are not tested before their re-location. The lack of studies on seropositivity in dog populations living in Mykonos Island does not allow a comparison of the prevalence between species. However, *L. infantum* is widely distributed in canine populations of Greece, and seroprevalence rates in regions of continental Greece range from 2% up to 50.2% (48, 49). An overall prevalence of 6.5% emerged in a recent serological and molecular study carried out on the Islands of Ios, Santorini, Tinos, and Skiathos (50). The prevalence of *L. infantum* in Greece and its presence in surrounding islands suggest that this protozoan most likely circulates also among canine populations of Mykonos.

Animals living outdoors are considered at high risk of infection with arthropod-transmitted pathogens, including *L. infantum* (51–53). Nevertheless, a significant correlation between outdoor lifestyle and the presence of *L. infantum* antibodies in the here examined cats was not observed. This is consistent with the results of a recent study, where 4/5 seropositive cats had, unexpectedly, an indoor lifestyle (27). Therefore, the present data suggest that indoor housing is not a sufficient preventative measure against leishmaniosis.

On the whole, this study confirms that cats living in the examined touristic areas of the Mediterranean basin are exposed to *L. infantum*. Infection rates in cats are generally lower than in dogs and the role of cats in the epidemiology of zoonotic leishmaniosis is still controversial and should be further investigated (16, 54). Recent findings have ultimately confirmed that dogs are better reservoir and spreaders of *L. infantum* than cats because under the same exposure conditions they display higher parasitic load, a determining factor for infectivity to sandflies (40, 55). Nonetheless, the role of cats as persistent source of infection for vectors (54) should not be overlooked, as they may amplify infection chances for dogs and, subsequently, for people.

In conclusion, the awareness toward feline infections should be kept at higher levels. Further studies evaluating the occurrence of *L. infantum* both in cats and dogs living in touristic destinations like Mykonos and Giglio Islands are warranted. Also, it would be interesting to conduct molecular studies to evaluate if species other than *L. infantum* occur in animal populations living in Mediterranean touristic spots. The importance of a constant surveillance is underlined by the fact that leishmaniosis has been regarded as an emerging threat in travelers (56) and cases of human leishmaniosis in tourists that visited Italy and Greece have been documented (57, 58). As routine examinations for *Leishmania* infection and application of chemoprophylactic measures in dogs and cats traveling with their owners are not regulated by laws, the establishment of standard preventative protocols for traveling animals is of importance for limiting the spread of zoonotic leishmaniosis and to minimize Public Health risks.

## Data Availability Statement

The datasets presented in this article are not readily available because they are represented only by the number of seropositive/seronegative cats in each study areas. Requests to access the datasets should be directed to smorelli@unite.it.

## Ethics Statement

Ethical review and approval was not required for the animal study because Blood samples were collected from cats upon a written consent obtained from the animal owners or the local municipality authorities and animal rights association authorities, in the framework of animal health screening programmes. Written informed consent was obtained from the owners for the participation of their animals in this study.

## Author Contributions

DT has supervised the whole study and drafted the manuscript. SM, MC, and AD participated in all project activities and in drafting the manuscript, after critically revising it. DD, AB, ADC, IR, RS, and BP have participated in the field, laboratory work, and in drafting the manuscript in various ways. All authors contributed to the article and approved the submitted version.

## Conflict of Interest

The authors declare that the research was conducted in the absence of any commercial or financial relationships that could be construed as a potential conflict of interest.

## Footnotes

^1^http://www.leishvet.org/fact-sheet-feline-leishmaniosis/feline-leishmaniosis-clinical-diagnosis/

^2^https://www.traghetti-giglio.it/en/tp-magazine/dog-friendly-holiday-giglio-island/

^3^www.mykonosanimals.org

## Abbreviations

VBD: Vector-borne Diseases
IFAT: Immunofluorescence Antibody Test
CI: Confidence Interval.
